# The Meanings and Prospects of Primo Vascular System from the Viewpoint of Historical Context

**DOI:** 10.1155/2013/439508

**Published:** 2013-05-16

**Authors:** Jongwook Jeon, Sanghun Lee

**Affiliations:** Acupuncture, Moxibustion, Meridian Research Group, Korea Institute of Oriental Medicine, 1672 Yuseong-daero, Yuseong-gu, Daejeon 305-811, Republic of Korea

## Abstract

The aim of this overview is to evaluate the primo vascular system research in the context of the history of meridian theory and the modern meanings of it. The 12 meridian systems were naturally presupposed in the conventional study of the meridians and acupuncture. But the excavations of Mawang-tui old documents and Sichuan Mianyang wooden puppet revealed the primordial concepts of meridians uncolored by the numerological cosmology of Han era. Further, the meridian map of horse, cow and hawk show another resemblance to the primordial type of meridians. Modern meridian theory has been challenged by the material based scientific theory and the primo vascular theory presents the most radical answer for it. It aims to reveal the anatomical entity of meridians. However, the study of primo vascular system is unexpectedly opening the new horizon of scientific integration of East and West beyond the mere searching for anatomical entity of meridians. Conclusions we have drawn from the historical reviews are, (1) the surface structure of the body reflects the physiopathological changes of inside the body, (2) by stimulating specific sites on the surface, it is possible to acquire therapeutic effects of certain symptoms, and (3) numbers and locations of meridian acupoints are variable among traditional meridian theories.

## 1. Introduction

Bonghan Kim, a medical scientist in Democratic People's Republic of Korea, was announced to identify the anatomical entities corresponding to the meridian system of Oriental medicine in 1963 [1]. It was known as the discovery of a third circulatory system and received worldwide attention at the time. Though following experiments from several countries was deployed, the original paper contains no detailed information about the process of the experiment. So the verifying experiments were very difficult to reproduce. After Bonghan Kim's sudden deposition and death, the authorities' strict closure of information was overthrown and it remained forgotten history [2].

Meanwhile, in 2002, Professor Kwangseop Soh in the Republic of Korea, performed reexcavation research to the reality of the meridian systems to revive Bonghan's achievements using advanced experimental apparatus. He newly named primo vascular system for Bonghan's system to express more fundamental system than others. At the end of 10 years' effort to identify the reality of meridian system, he held the first international primo vascular conference with leading scholars of the field. In this paper, we briefly review historical aspect of meridian systems which had been presupposed in the system of Bonghan Kim or Professor Kwangseop Soh. After Bonghan Kim announced that he had discovered the anatomical entities of the meridian system, meaningful historical excavations were performed showing a prototype of the meridian systems quite different from the conventional. And secondly, the stark dichotomy between Oriental medicine and Western medicine is being challenged on multiple layers of readjustment by the contemporary atmosphere of fusion research [3]. Only after considering seriously these two apparent situations, we can open a wider horizon for future researches and make constructive debates for the primo vascular system (or Bonghan system).

## 2. Mawang-Tui Old Documents and Mianyang Wooden Puppet Unearthed

**Figure 1:**
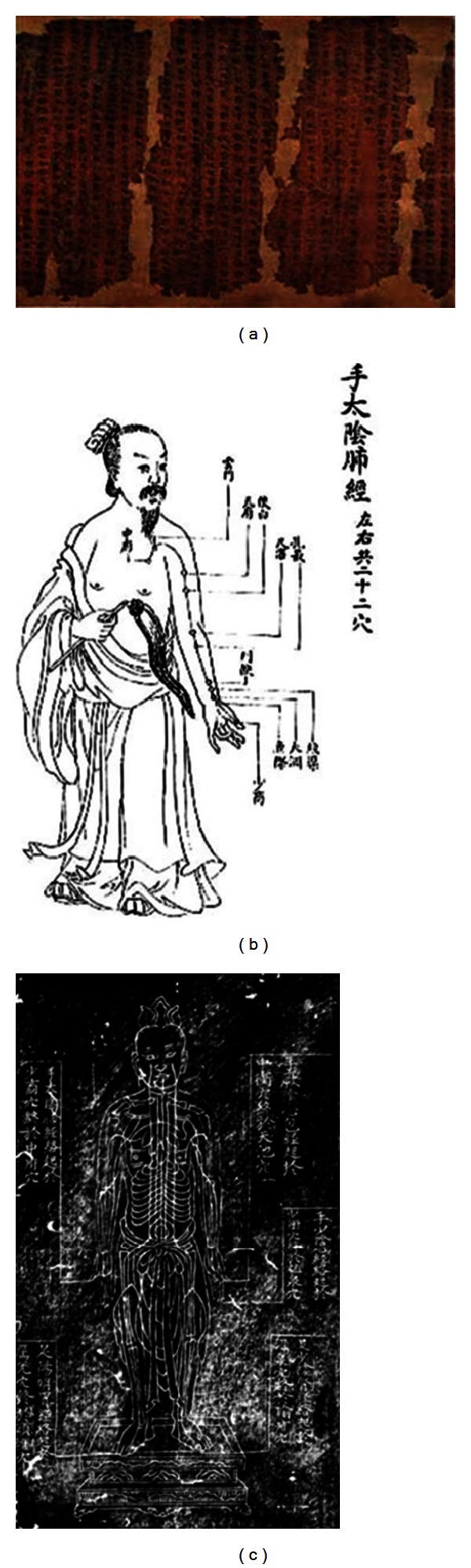
(a) Mawang-tui old documents on medicine. It was described on Yin-Yang eleven meridians for moxibustion and Foot-Arm eleven meridians for moxibustion. (b) Drawings of traditional meridians (part). (c) Meridians carved in stone in Ming dynasy, preserved in royal library in Japan (*明正統石刻銅人经脉图*, *日本宫內厅藏*).

It is well known that the present twelve line meridian system had been based on Huang Di Nei Jing (The Yellow Emperor's Internal Medicine; *黄帝內经*) which was established in Han empire era (B.C. 206~A.D. 220) and made a great influence on the literature of Oriental medicine for 2000 years. But about 200 years earlier than Huang Di Nei Jing, a remarkable excavation of old documents was made in the Mawang-tui [4]. Fourteen kinds of the old documents contained medicinal resource, and 2 kinds of them described old type of meridian system (Figure 1(a)). Unearthed in 1973, the old documents revealed that it was quite different from that of Huang Di Nei Jing (Figure 1(b)) or other later one (Figure 1(c)). Admittedly, these Mawang-tui old documents had been made around B.C. 168 delivering direct information of medicine of the time, such as “Yin-Yang eleven meridians for moxibustion” and “Foot-Arm eleven meridians for moxibustion.” Considerable researches have been made in the relevant academia, so a brief conclusion widely recognized would be helpful. Characteristics common to the two kinds of meridians on Mawnag-tui old documents are just like the followings [5, 6].

(1) Meridians are mainly used for the moxibustion and pyaemia emissions (biensi, *砭石*) therapy rather than needling acupuncture therapy.
(2) There are only descriptions of meridian line but none about the acupoints.
(3) Number of meridians are not twelve but eleven.
(4) Among the eleven, only 2 or 3 have connections between meridians and viscera.
(5) Meridians are independent each other, not like the unified circulatory system consisting of 12 meridians in Huang Di Nei Jing.

Though there are more detailed differences between them, I want to check these five things first. Distinctions become clear by these five points. Conventional concept of meridians is a certain energy (qi, *氣*) route that consisted of acupoints which are certain places under the skin for acupuncture therapy. But in Mawang-tui old documents acupoints (*穴*) were not present in the meridian pathway, and the meridian itself was not for acupuncture but for moxibustion therapy or pyaemia emissions treatment. Number of meridians and the names are also different from conventional twelve meridians (Table 1). Primitive type of naming is seen such as the shoulder meridians (*肩脈*), ear meridians (*耳脈*), and tooth meridians (*齒脈*), which used particular parts of the body. Furthermore, it attempts only a couple of connections between organ and meridians, and the meridians are described as isolated lines independent of the others. It is just a connecting line between one part of the body and the other, an one-to-one-relation line. According to modern multidisciplinary researchers, the meridians in Mawang-tui old documents did indicate specific palpation site of human body rather than the long pathway of qi. So it is proposed that the pulsing signals of eleven meridian system of Mawang-tui were the prototype of the later twelve meridians diagnosing method [4]. Collectively, different from the present meridians, it is just a pulse site which indicates health status of body and a place for therapeutic treatment at the same time. 

**Figure 2:**
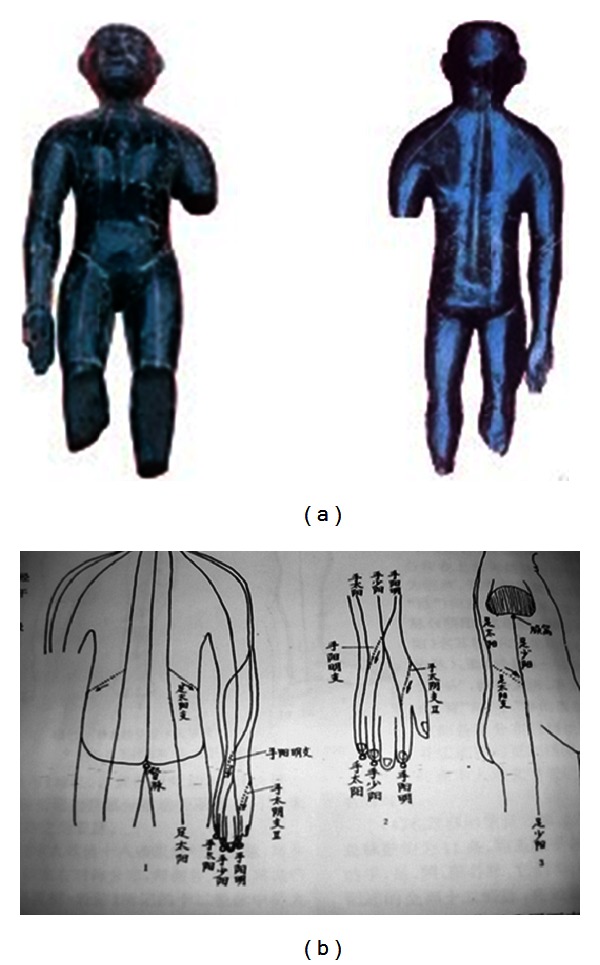
(a) Sichuan Mianyang lacquered wooden puppet. It is believed to be made in B.C. 179~B.C. 141, slightly later or contemporary to Mawang-tui. (b) There are ten meridians, no acupoints, with several connections of meridians.

Moreover, in 1993, a wooden puppet was unearthed in Mianyang City of Sichuan Province [7]. On this lacquer painted wooden puppet, drawings of human meridian path were found (Figure 2(a)). Compared with Mawang-tui old documents, it had the one meridian that Mawang-tui did not have at the moment and added the dorsal median meridian (*督脈*). Instead, the puppet lacks three yin meridians on the hand and also has no acupoints. There are connections between meridian lines which are faintly seen on the wooden surface (Figure 2(b)), and so this puppet is estimated to show an important step forward the whole unified concept of body, including the addition of the dorsal median meridian. It is the reason why this puppet is considered as an intermediate step of Mawang-tui and the following Huang Di Nei Jing [5, 8]. 

These excavated relics are shocking to the experiment researchers who had believed the theory of Huang Di Nei Jing. Where is the acupuncture meridian we know? 

## 3. The Numerological Cosmology of Han Empire and the Meridian Theory

Meanwhile, the following interpretations are gaining strength by the evidence collected along these excavations [4, 8, 9]. There had been a variety of therapeutic theories and practices in various areas around China, prior to gaining ultimate legitimacy. But with the establishment of the Han empire (B.C. 206~A.D. 220), the integration of each theory was rapidly accelerated in every field of thought and society. Yin-Yang and five elements theory as the representative convergence, affected by numerological cosmology of the Han empire, made huge influence on the following history. It is interpreted as systematic corresponding system in a modern version. The human body has come to share the verge of the law penetrating the entire universe, which means not only the surrounding nature (mountains, rivers) but also the operation of the national management system (bureaucracy), the flow of economic products, and the movements of heavenly bodies [9]. From the initial clue of connection between rustic old meridian and a couple of viscera, a full-fledged systematic corresponding system of twelve viscera/entrails and twelve meridians were completed finally. Since then, a systematic corresponding system of the Han empire lasted for two thousand years without major change at least in acupuncture meridian theory. But in a more advanced interpretation, it seems that Mawang-tui meridian system preserves the original meanings of meridians, not wholly colored by the unified concept of Yin-Yang Wu-Xing (*陰陽五行*) cosmology, which is later invention of numerological intellectuals of Han empire [8].

In Oriental medicine twelve meridian theory based on Yin-Yang Wu-Xing has got the self-consistency and metaphysical power for long time [9]. It is accepted now as the core of the Oriental medicine. Still in many clinics using Oriental medicine, they show successful treatments for patients with the concept of qi-flowing meridians. However, it is not sufficient. It must be reminded that the mainstream treatments should be grounded on the explanatory framework of our time of science [3].

## 4. Mixing of Practice and Theory in the History of Oriental Medicine 

 The paradigm of the Han empire so formed gives a significant impact on us until now. According to this paradigm, diseases come from an imbalance of yin and yang or interference with the circulation of the qi, and the four diagnostic methods are presented to catch the state: (1) visual inspection, (2) listening/smelling, (3) questioning, and (4) touching (mainly palpation). Thus obtained data are connected to each other in a regular pattern to understand the disease or symptoms [10]. This patternization of symptoms (bian zheng; *辨證*) in Oriental medicine has been argued for a long time because they cannot be understood as conventional scientific thought. Also treatments consisting of acupuncture and herbal medicine are linked to the bian-zheng (pattern of symptoms). It is like a synchronicity of the events of the world, in the modern terms. A disease occurs as a disharmony of Yin-Yang and five elements of the body because the universe is connected to each other with the concept of systematic correspondence. Oriental medicine has been characterized by this cosmology of Yin-Yang and five elements, so the medical experience and treatments were understood as functional perspective (i.e., systematic correspondence), which is strikingly different from the Western perspective (i.e., causality) [3, 9]. 

But is this integration of Han empire, as alleged, an immovably robust system? Or is it an incomplete, vulnerable one and needed to revise itself continuously? We are now forced to put stress on the latter; it had problems and cracks around the system from the beginning. In the first place, though focused much on the theoretical tidiness or completion of twelve meridian systems, there had been little evidence based on clinical effectiveness [6]. In fact, when acupoints have been added over a hundred of new comers until 361, for example, expansion criteria of the number of acupoints, their major effectiveness to certain symptoms was never discussed or explained [6]. And later, that the dorsal median meridian (*dumai*, *督脈*) takes two branches is never explained but just described [6]. This important addition or revision has no specific theoretical explanation or clinical evidence by responsible writers or doctors. This shows an inevitable weakness of meridian theory based on the mainstream ideology of the Han empire, and now we need new methodology to organize the new findings and experiences in a modern setting.

When it comes to herbal medicine, it is a more obvious failure. From the first full-scale pharmacology text, Herbal Medicine of Divine Farmer (*Shennong Bencao Jing* *神農本草經*), the systematic correspondence model could not harmoniously incorporate the various herbs with respect to Yin-Yang and five elements, rather just using the scale of hot or cold using Yin-Yang only [9]. By the 12th century, four great physicians of Jin and Yuan dynasties (*金元四大家*) attempted a meaningful try of integration, but also to an incomplete and sloppy end [9]. It means the cosmological integration of herbal medicine in Oriental medicine was incomplete, so it was vulnerable to other competitive cosmologies through the history. Therefore it seems natural that herbal medicine might easily be disseminated by the Western methodology, especially by pharmacology. 

 In respect to acupuncture meridian theory, Mawang-tui discovery itself reveals the distinct process of changes of the meridian theory. It features a rustic anatomical view based on actual observations, just a blood vessel, and in a respect shows mechanical view of what looked to be in their senses. In consensus of the field, it was described that the Mawang-tui meridians (*脈*) have three complex meanings [8].

(1) Meridian (mai, *脈*) was blood vessels in the original sense.
(2) Meridian (mai, *脈*) was Pulsation in the own character. In diagnostic process, they could pick out other forms of Pulsation in comparison with the normal pulsation.
(3) Meridian (mai, *脈*) was also a treatment site to achieve a therapeutic effect by stimulations including moxibustion initially or acupuncture and both later. These three aspects of the meanings of meridians have been changed through the history until now (Table 2).

## 5. Primo Vascular System on the Dynamic Rebuilding of Oriental Medicine

 A number of multifaceted researches have been performed to identify meridians and to explain them anatomically. Spotlighted are the recent active researches using fMRI equipment. Their conclusions are that the effect of acupuncture relies a significant portion on neurotransmitter systems. Though this neural hypothesis that the peripheral and central nervous systems play an important role in acupuncture effect is getting scientific basis for defining meridians [11], others oppose this interpretation. They contend that effect of acupuncture appears after a certain period of time and that acoustic shear wave in the tissue of human body fits well with both the effect of acupuncture and the image of the meridian [12]. Both have considerable support and experimental evidence. Overall the effect of acupuncture might encompass these phenomena. But these experimental setups presuppose that meridian system should be understood via other structures of human body. In respect to that point, primo vascular system seems to provide a very unique one unlike others. Researchers on primo vascular system just focus on revealing the exact factual structure, which has never been presented to us by visual sense, a new anatomical circulatory system [13].

**Figure 3:**
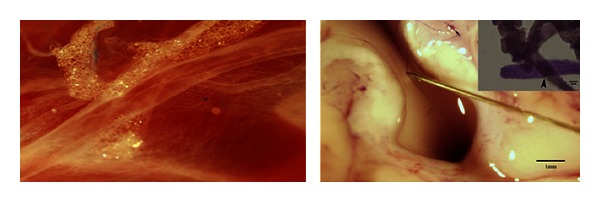
Bonghan duct (primo vessel) floating in lymphatic vessel of rabbit (Lt) and rabbit ventricle (Rt). Bonghan duct is increasingly recognized as the anatomical entity of acupuncture meridians. It runs every part of living body both in human and other vertebrate animals.

They try to show or explain a tube (with the diameter of 10~30 *μ*m) connecting the whole body which corresponds to the meridians or corpuscles which correspond to the acupoints, independent of the other circulatory (vascular or lymph) systems or nerve systems. In this point primo vascular system researchers authentically follows the study of North Korea's Bonghan Kim [2, 14]. They are trying to demonstrate the structure and function of the primo vascular system in terms of sophisticated modern terminology based on various animal experiment, daring in front of the establishment of physiological knowledge [15–20]. In particular, Bonghan Kim argued that Bonghan duct can be found in every vertebrate in common [1] (Figure 3), which fits with the long tradition of acupuncture treatment on the diseases of horse and cattle, and hawk for hunting [21]. And interestingly, the meridians of these animals are more similar to those of Mawang-tui or Mianyang wooden puppet. It is highly necessary to take a close look on the remaining prototypes of meridians.

**Figure 4:**
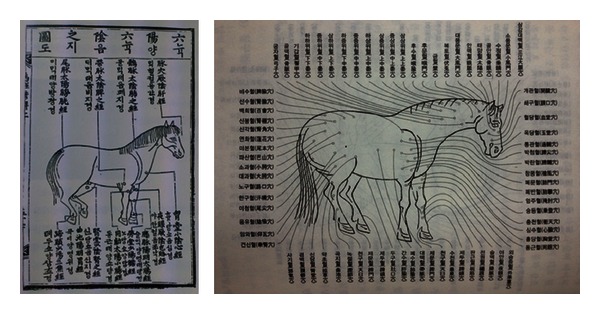
Lt. Drawing of meridians of a horse in the book *Newly Edited Horse Disease Therapy Regimen* in 1399, early Joseon dynasty. The meridians are a common phenomenon in vertebrates. Rt. Acupoints of horse with relation to meridians. Meridian names do have one of the name of its major acupoints.

It is interesting that the twelve meridians of horse have their main acupoint name at the head of names (Figure 4).

It may be another support that the meridians first served as diagnostic and therapeutic sites. Also it seemed to start first not as a long line of acupoints with similar functions but as a relevant place to other parts of horse body, likely to the inner organs. Moreover, there remains “theory on the relation of spot to pain (*dian-tung lun* *點痛論*)” which presents 45 specific signs or movements with the pain sites of the horse [21]. Several examples are as follows.

(1) Walking with straight legs is the sign of pain of the upper knee.
(2) Not moving with the head up is because of the pain in the hoofs.
(3) Walking with head nodding is because of the pain of hind limbs.
(4) Walking with head shaking is because of shoulder pain.
(5) Poor moving of the hind legs is because of kidney pain.
(6) Choppy breathing is due to the pain of the lung meridian.
(7) Urgent wake-up and urgent lie-down is due to the pain of spleen meridian.
(8) Walking with upraised tail is due to the pain of large intestine.
(9) Walking with rolled tail is due to the pain of small intestine.

We can compare the fragmental knowledge of diagnoses and therapies with the original notions of meridians in the Mawang-tui and Mianyang wooden puppet. With the accumulation of evidence of site-to-site relations of the body, the generalized concept of interconnected meridians, such as viscera-limbs, inner-outer, and upper-lower, might have its mature conditions. Though there are multiple dimensions on the way of generalization, we do consider that these pieces of evidence are worth of advanced investigation in the course of meridian research. 

This is the time to consider whether the primo vessel (or Bonghan duct) is the anatomical structure of the meridians. Though tentative, we are in the negative position when we see in line with the history of meridian theories and the remaining fragmental knowledge about the vertebrates. It sounds reasonable saying that anatomical structures of meridians are more reliable in the old documents of Mawang-tui in the respect of reality, because the meridian theory of Huang Di Nei Jing had been deeply colored by theoretical cosmology, that is, a kind of ideological generalization in Han empire era [9]. After wards, twelve meridian theory of the Huang Di Nei Jing was neither physically based nor fully explained until the recent day. Meridians are more of a cosmological image than a factual reality, though the pulsation-organ relationship remains alive in part [6].

Some primo vascular system researchers recently do not just try to meet it with the traditional meridian theory along with these notions. Further they try to reveal that the relationship of stem cells, new function of DNA, and the possibility of cell therapy are main concerns of the field [22–24]. 

**Figure 5:**
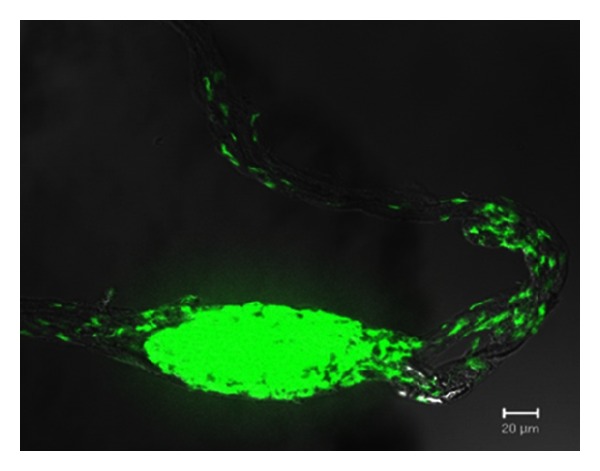
Sanal cells with dense DNA appear bright green on fluorescence staining in bovine heart corpuscle (visible part).

 Even the meridian-oriented informing system is also a range of research areas expanding, especially in that Sanal matures when it receives light, and Bonghan duct has features of bundle structure, which reflects the characteristic of plants (Figure 5). Some researchers contend that the primo vascular tube is regarded as fiber-optic cable with high-speed flow inside of it. Like Western medicine is backed upon medical physics, this new medicine might be grounded on electromagnetic power, light, and biophoton which is detectable by photo-multiplier tube [25]. 

On the other hand, as a unique part in Oriental medicine, meditation and respiration training may have an impact on the circulation of the fluid with rhythmical stimulation of Bonghan duct in the multiple layer of abdominal fascia [26]. During the respiration, respiratory-related effect on primo vascular system is expected in the rhythmic contraction and relaxation along with continuous peritoneal muscle activity.

**Figure 6:**
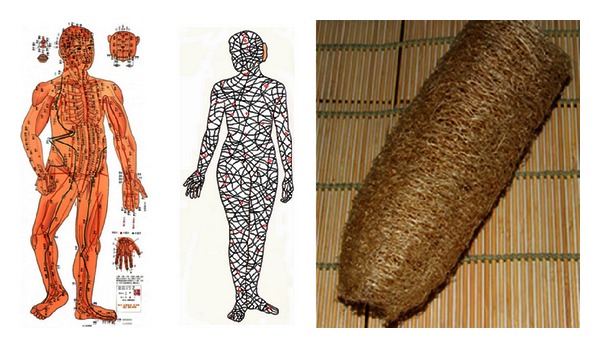
Loofah sponge model of primo vascular system (Bonghan-Fascia Model, Lee and Soh, 2009 and 2011).

Lee Byung-cheon, researcher of primo vascular system, presented a new generalized model of more than 10 years of experimental research on primo vascular system. He shows a sponge model of human body which well represents the primo vessel running in and outside of the blood vessels around the body, in and outside of the organs, and all the musculoskeletal or connective tissues like a loofah sponge (Figure 6) [27, 28]. This is estimated to provide the imaginative basis of a new research direction on the network of primo vascular pathway and on the mechanisms of the regeneration of wounded tissue [22, 29]. Interestingly, this model has common aspects with the author of Energy Medicine, James L. Oschman, who suggests that the meridians penetrate all the organs of the human body, including cells and cellular organelles [30]. 

Thus the most promising area of primo vascular system's future development might be the research of self-organized circulatory system that encompasses the energy and information. This means that they are trying to answer on the role of the primo vascular system in respect to developmental, healing, and regenerating functions to which modern medicine is most vulnerable [31]. 

## 6. Discussion

In the ancient Chinese book Mencius is stated, “What is inside your body, necessarily disclose itself (*有諸內形諸外*)” [32]. In the book Great learning is stated, “When others easily watch me like looking into the five viscera, it is useless trying to hide (*人之視己如見其肺肝然則何益矣*)” [33]. This way of thinking seems deeply rooted among ancient Chinese. And it is likely that the ancient Chinese thought that it was very easy to look inside the body because the inner side of human body must be throwing a signal to some areas outside of the body. It has been identified in the human body, so to speak, “theory of three sites and nine diagnoses (*三部九候論*)”, and nine pulsation sites of the surface of human body were conceived to regulate all the problems of human body like remote-controller [8]. And these original concepts of human body were systematically combined with numerological cosmology which unified the body and the universe with systematic correspondence mainly of the Han empire.

Could the 12 meridians be regarded as ultimate anatomical system of Oriental medicine? On which empirical evidence had the theory been established? We recall again the historical fact that Bonghan's theory was released in 1963. It is obvious that Bonghan Kim had in his mind the conventional 12 meridian system as anatomical entities known at that time. However, Mawang-tui old documents were excavated in 1973. Newly found old documents strikingly influenced almost all the relevant fields of academia. In Mawang-tui the meridians of the human body were not 12 but 11 without acupoints. Twenty years later in the mountains of Sichuan Mianyang province lacquered puppet was excavated with meridians drawn on the surface of the body and 10 meridians are identified. The discovery of the two was a big turning point for researchers especially in medical history. Until then it was accepted without a doubt that there had been acupoints first, and later the meridian line was formed by the grouping of similar acupoints. Those excavations, however, transformed the old concepts dramatically. In addition, the formation of the 12 meridian system seems to have been influenced by various routes and practices, and it is increasingly admitted that we should carefully winnow sophisticated historical literature to understand the true empirical phenomena of meridians [4]. Specifically, beyond the conventional study, we first should separate the reality of meridians (pulsation, diagnosis, and treatment) from the philosophical structures (numerology, philosophy of systematic correspondence). A newly refined model of meridians should be presented, adapted to the scientific progress in the 21st century. This is why the current researchers on meridians also need to be aware of the advanced knowledge from historical excavation.

Does the primo vascular system belong to Oriental medicine or Western medicine? The answer indulges in a dilemma because the definition of the term already has various deflects. Oriental medicine persistently made an attempt to mangle various disparate elements or practices from its departure, and it is such a dynamic structure allowing active influx, acceptance, and generalization with all the contradictions, inconsistencies, eventually to a different way of systematization or reconstruction. The case of New York Times journalist Reston, in 1971, and “auricular acupuncture” is representative examples. Western medicine also openly applied Galen's theory of fluids in the clinics until modern medicine methodology was established by Pasteur and Koch [10]. When considering that Western medicine and Oriental medicine also made their way with a ceaseless acceptance and reconfiguration of heterogeneous elements, and due to the imperfect explanatory framework, the effort of modification and systematization is still under process; the primo vascular system in its present form might be the most fierce contact and conversation between the two medical systems [3]. 

The target of Oriental medicine or Western medicine is the same “human body.” However, both emphasize the heterogeneity, respectively, focusing on the usefulness of each approach which is either holistic or analytical. Maintaining a dichotomic view that human body is divided into matter and energy, they cause frictions in terms of qi (*气*) and meridians whether they should be defined as a tangible presence or just a functional phenomenon. However, when you look back on the process of formation of the initial meridian theory, we cannot deny blood vessels and blood circulation in substance had been recognized as the meridians. Surely meridian theory was formed by combining clinical experience of the specific effect of certain stimuli on existential objects and theorized complying on the zeitgeist of ancient Han era. After that, even though there are some changes historically in meridian theories, the consistent thought in meridian theories to control diseases by stimulation of the body surface has been unchanged. This might be served as a guideline primo vascular system researchers can consider with all the differences of meridian systems in history, that is, number of the meridians, the location of acupuncture points, the pathways of meridians, and connections between the meridians and the viscera. Though primo vascular system is a powerful theory to explain the mechanism and the entity of the meridians, the conventional meridian theories were considerably affected by the spirit of the times. So it is required that we should clarify the core meanings of meridians from other cultural products. From those analyses we can derive free and creative premises for the primo vascular researches, which may be a completely new modern meridian theory. We expect that it may be the starting point that reflects the modern zeitgeist not only integrating the matter and spirit but also connecting the Eastern and Western perspective of the human body.

## 7. Conclusion

Based on the historical research on the old documents and practices, the implicit premises of conventional meridian studies should be modulated with a certain transformation. Though Bonghan's theory was set out clearly away with previous conventional idea, it was under the impact of long-standing tradition of meridian theory. But we think primo vascular system was apparently sparked by the meridian theory in Oriental medicine; the results might be a completely new intellectual discovery never known to us. It is increasingly likely to act as a prelude to mobilize a new medicine in the disciplines of all ages and countries, which belongs neither to Western nor to Oriental. We expect that it is just a probable vision upon the historical perspective of Oriental medicine.

## Figures and Tables

**Table 1:** Names of meridians in Mawang-tui and Mianyang in comparison with Huang Di Nei Jing. Arrows show historical changes of meridian or equivalent names from Mawang-tui to Huang Di Nei Jing. Red arrows indicate no equivalent names in Mianyang wooden puppet.

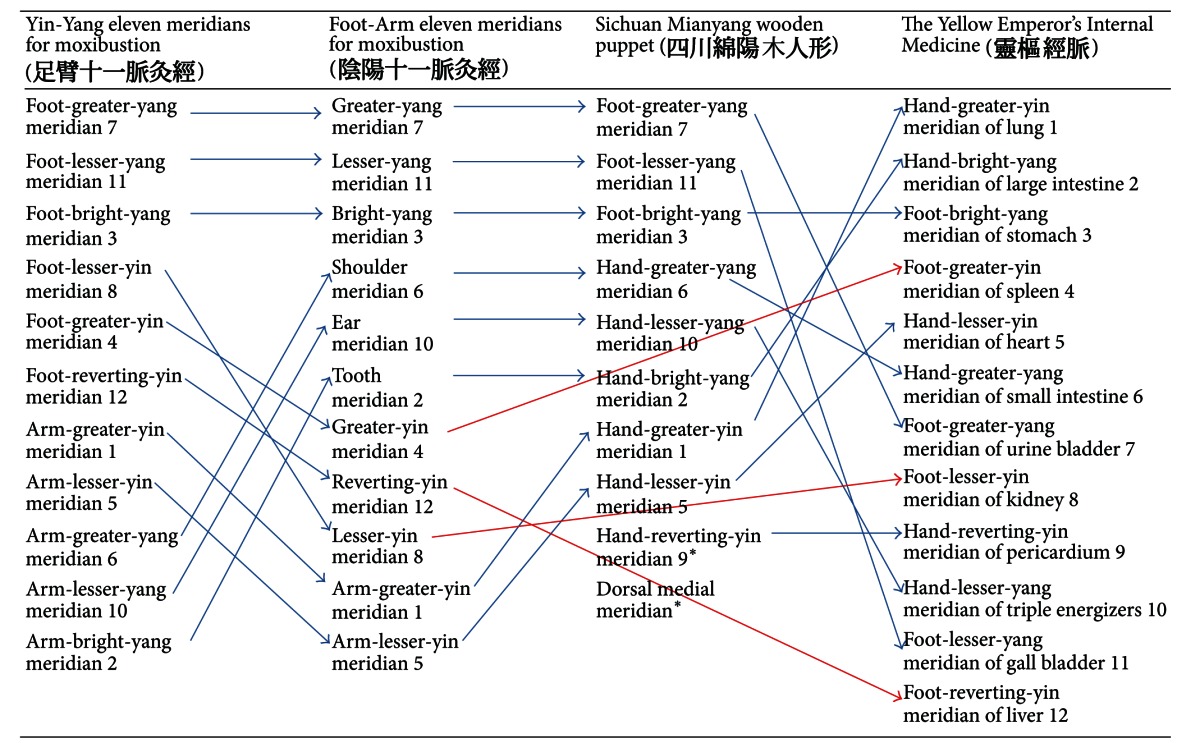

*Denotes the meridian only appeared in Mianyang wooden puppet.

**Table 2:** Primo vascular system in comparison with meridians in the history of Oriental medicine.

	Mawang-tui documents (B.C. 168)	Sichuan Mianyang puppet (B.C. 170~140)	Shiji by Sima Qian (B.C. 109)	Huang Di Nei Jing (A.D. 100?)	Bonghan system (1963)	Primo vascular system (2002~)
Primary meridians (number & type)	11 line No AP	10 line No AP	12 line? AP	12 line AP	12 line? BD/BC	Sponge-like form BD/BC

Diagnostics	Pulse	?	Pulse	Pulse	?	?

Therapeutics (Local & systemic)	M, PE	A?	A M?	A, M	A, EA	A, EA

AP: acupoints; PE: pyaemia emissions; A: acupuncture; M: moxibustion; EA: electroacupuncture; BD: bonghan duct; BC: bonghan corpuscle.
